# Shear-wave elastography assessment of gluteal muscle contracture: Three case reports

**DOI:** 10.1097/MD.0000000000013071

**Published:** 2018-11-02

**Authors:** Ruiqian Guo, Xi Xiang, Li Qiu

**Affiliations:** Department of Ultrasound, West China Hospital of Sichuan University, Chengdu, Sichuan, China.

**Keywords:** gluteal muscle contracture, musculoskeletal imaging, ultrasonic elastography

## Abstract

**Rationale:**

Gluteal muscle contracture (GMC) is a clinical syndrome characterized by limited hip joint function due to fibrosis and contracture of the gluteal muscle and fascia fiber. Imaging examination is significant for its diagnosis and guidance of surgical treatment.

**Patient concerns:**

This report presents 3 cases of GMC with bilateral involvement and summarizes the literature on the imaging findings of this disease. The clinical symptoms and presentations of the 3 cases well-matched the clinical diagnosis of GMC. Preoperative ultrasonography including elastography was performed. To our knowledge, studies based on the elastography findings of the disease are limited.

**Diagnoses:**

The diagnosis of GMC was finally confirmed by postoperative pathologic examination.

**Interventions:**

All 3 patients then have a surgical therapy which cut off the contracture tract.

**Outcome:**

Symptoms of abnormal gait and limited hip joint function were greatly improved after surgical treatment.

**Lessons:**

The elasticity of the GMC zone in patients with GMC is higher than that of the muscles in the corresponding sites of healthy people.

## 1 Introduction

Gluteal muscle contracture (GMC) is a clinical syndrome characterized by limited joint function due to the fibrosis and contracture of gluteal muscle and fascia fiber. Patients with GMC typically present with abducted and externally rotated hip and are unable to bring both knees together when squatting. The first case of GMC was reported by De Valderrama in 1970.^[1]^ Most patients were young children (6–18 years), and most lesions were secondary to repeated intramuscular injections in the buttocks.^[2–4]^ A number of reports about GMC have been published thus far, but its etiology, pathogenesis, classification, and treatment remain controversial.^[5–7]^ Preoperative determination of the extent and severity of the lesion helps the clinician to design the optimal operation scheme. Therefore, imaging is important in preoperative examinations. Imaging report on GMC is rare, and ultrasonic elastic examination of the disease has not been reported yet.

Shear-wave elastography (SWE) is an exciting and rapidly evolving ultrasound (US) technique allowing quantitative evaluation of soft-tissue elasticity. Therefore, this study aimed to present the clinical manifestations, pathology, and elastograms of 3 patients with GMC, which were measured by US, and review the literature on the imaging findings of GMC.

## 2 Method

This report presented a series of 3 cases of GMC. Obtaining approval from an ethics committee or institutional review board approval was not required. However, written informed consent was obtained from the 3 patients for publication of this case report and accompanying images.

In this study, the US equipment Aixplorer (SuperSonic Imaging, Aixen-Provence, France) with a 4 to 15 MHz or 2 to 10 MHz probe was used depending on the depth of the gluteus maximus muscle. The shear-wave velocity was selected to reflect the stiffness of the muscle because the muscle is an anisotropic tissue. Details of the 3 cases are presented in the following sections.

## 3 Case presentation

### 3.1 Case 1

#### 3.1.1 Clinical characteristics

An 8-year-old boy visited the hospital for a history of out-toeing gait and abnormal frog-leg sitting position. On examination, both lower extremities were outwardly rotated and both knees could not be put together when standing. When sitting, he could not cross or overlap his legs. When squatting, both lower limbs demonstrated a type “o” feature and the heels did not touch the ground. The muscles on both sides of the buttocks were noticeably tight. The flattening of the right buttock was also observed. Bilateral hip adduction and abduction activities were significantly limited. The angle of passive adduction in hip flexion was −40°.

#### 3.1.2 Ultrasonography

##### 3.1.2.1 Gray-scale ultrasound and color Doppler flow imaging

**Figure 1:**
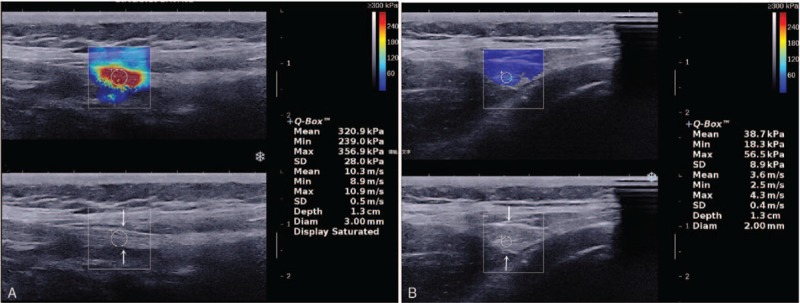
An 8-year-old boy with gluteal muscle contracture. (A) Longitudinal view of the gluteus maximus muscle. The hyperechoic contracture strip can be identified easily (arrows). Its color-coded shear-wave elastogram was mainly orange-red. (B) Transverse view of the gluteus maximus muscle. Gray-scale ultrasound image clearly shows the strip (arrows). The color-coded shear-wave elastogram of the contracture strip was blue.

Gray-scale ultrasound (GSUS) showed bilateral gluteus maximus muscle thinning and strips of echogenic foci (contracture strips) inside the muscles, within which no significant blood signal was observed using color Doppler flow imaging (CDFI). The right strip was approximately 5 mm thick, and the left was approximately 6.6 mm thick (Fig. 1A and B).

##### 3.1.2.2 Shear-wave elastography

The SWE color-coded elastogram of the contracture zone was mainly orange-red in the longitudinal section and blue in the transverse section. The average of the mean shear-wave velocity was 9.15 and 3.10 m/s, respectively (Fig. 1C and D).

### 3.2 Case 2

#### 3.2.1 Clinical characteristics

A 10-year-old girl was found to have an abnormal gait 1 year ago. On examination, she could stand with knees put together. When sitting, she could not overlap her legs. During squatting, both knees are rounded, and snapping sound could be heard from the sliding contracture bands. The bilateral gluteal muscles appeared to be slightly tensed upon palpation. Bilateral hip adduction and abduction activities were mildly limited. The angle of passive adduction in hip flexion was 0°.

#### 3.2.2 Ultrasonography

##### 3.2.2.1 GSUS and CDFI

**Figure 2:**
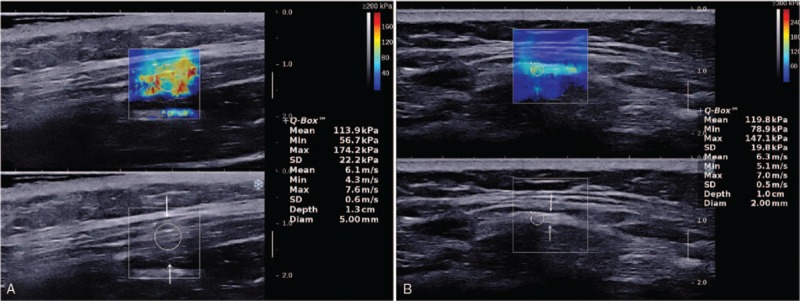
A 10-year-old girl with gluteal muscle contracture. The strip was indicated by arrows. (A) Longitudinal view of the gluteus maximus muscle. The color-coded shear-wave elastogram of the strip was uneven cyan, orange, and red. (B) Transverse view of the gluteus maximus muscle. The color-coded shear-wave elastogram of the strip is uneven cyan.

Bilateral gluteus maximus muscle thinning and intramuscular strips of echogenic foci (contracture strips) were observed; the strips on the right and left sides were about 4.8 and 6.5 mm thick, respectively. No obvious blood flow signal was observed (Fig. 2A and B).

##### 3.2.2.2 Ultrasonic elastography

The color-coded shear-wave elastogram was uneven cyan and orange in the longitudinal section and uneven cyan in the transverse section, and the average of the mean shear-wave velocity was 5.84 and 4.12 m/s, respectively (Fig. 2C and D).

### 3.3 Case 3

#### 3.3.1 Clinical characteristics

A 28-year-old woman was unable to walk in a straight line. On examination, her knees could be put together when standing. She could not cross legs in sitting postures and bring knees together during squatting. The bilateral gluteal muscles appeared to be moderately tensed upon palpation. Bilateral hip adduction and abduction activities were moderately restricted. The angle of passive adduction in hip flexion was −10°.

#### 3.3.2 Ultrasonography

##### 3.3.2.1 GSUS and CDFI

**Figure 3:**
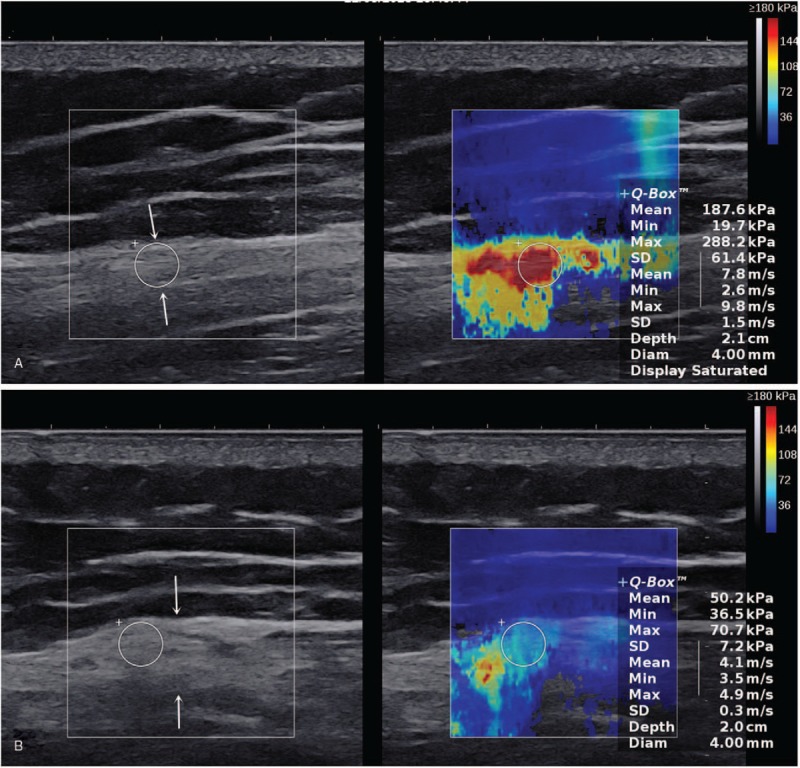
A 28-year-old woman with gluteal muscle contracture. This figure shows the gray-scale ultrasound image and shear-wave elastogram of the contracture strip (arrows). (A) Longitudinal view of the gluteus maximus muscle. (B) Transverse view of the gluteus maximus muscle.

The US revealed bilateral gluteus maximus muscle thinning and intramuscular cord-like zone of strong echo. The thickness of the strong echo zone was about 12 mm on the left side and about 7.4 mm on the right side. No significant blood flow signal was observed (Fig. 3A and B).

##### 3.3.2.2 Shear-wave elastography

In the longitudinal section, the SWE color-coded elastogram of the contracture zone was uneven cyan, orange, and red. The average of the mean shear-wave velocity was 7.18 m/s (Fig. 3C). Correspondingly, the color-coded shear-wave elastogram of the contracture zone was uneven cyan and the average of the mean shear-wave velocity was 4.13 m/s in the transverse section (Fig. 3D).

#### 3.3.3 Pathology

The 3 patients had no history of any treatment and selected surgical therapy. Besides the bilateral gluteus maximus muscles, the gross pathologic examination of the 3 patients also revealed piriformis muscle contracture and fiber plate-like changes. Pathology confirmed the diagnosis of GMC. Symptoms of abnormal gait and limited hip joint function were greatly improved after surgical treatment. The average follow-up period was 3 months, We found no postoperative complications.

### 3.4 SWE of non-GMC muscles

**Figure 4:**
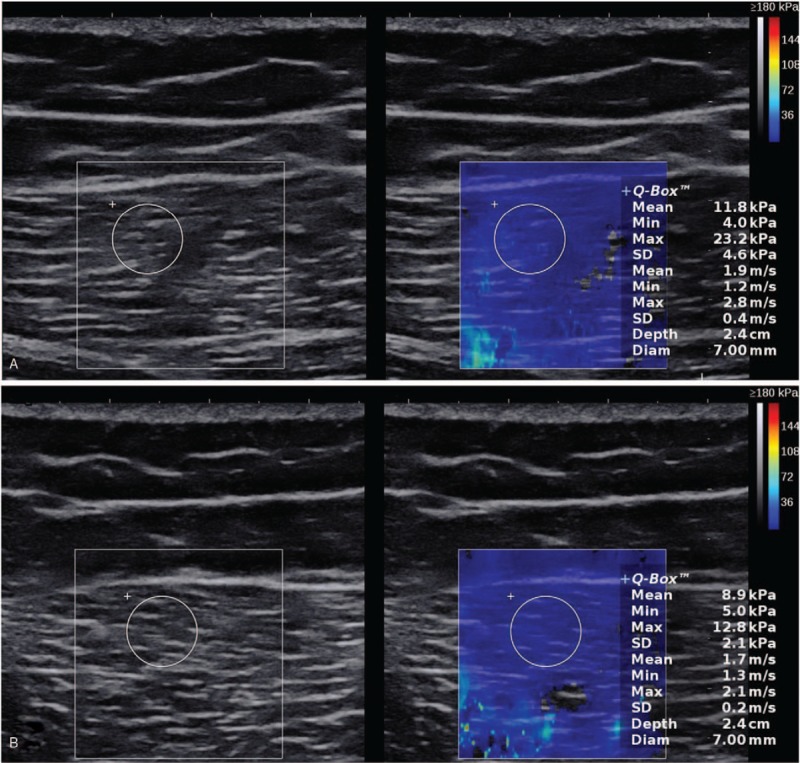
Ultrasound characteristics of nongluteal muscle contracture muscles. (A) Longitudinal view of the gluteus maximus muscle. (B) Transverse view of the gluteus maximus muscle.

Whether in the cross-section or the longitudinal section, the color-coded shear-wave elastogram was uniform and in blue-green (Fig. 4).

## 4 Discussion

Gluteal contracture is a clinical syndrome characterized by hip dysfunction due to fibrosis and contracture of the gluteal muscle and fascia fiber caused by various causes. GMC occurs worldwide,^[7]^ but it is highly prevalent in China with an overall childhood incidence rate of 1% to 2.5%,^[8,9]^ mainly involving school-aged children. Moreover, the younger the patients at the time of gluteal injection, the higher the prevalence. However, the etiology and pathogenesis of GMC are still controversial.^[5,6]^ Most authors believed that gluteal injection was the most common cause of the disease.^[2–4]^ Reports on genetics are few. All cases in this study had a history of repeated gluteal muscle injection and have no family history. Mechanical damage and inflammatory stimuli of chemical agents because of repeated gluteal injections caused hemorrhage, exudation, degeneration and necrosis, and proliferation of fibrous tissue, contributing to atrophy and fibrosis of gluteal muscle tissue and contraction of myofascial tissues. The clinical characteristic was limited flexion and adduction of the affected hip.^[10]^ GMC was usually bilateral, which might be related to the equal probability of injection on both sides. All 3 patients in this study had bilateral involvement.

The clinical symptoms and signs of GMC become more severe with age, and the incidence of complications, such as pelvic tilt and scoliosis, increases. Therefore, early diagnosis and treatment are closely related to the prognosis of patients. In the absence of imaging examinations, diagnosis is based on clinical manifestations, which often delays treatment, resulting in pelvic deformity, scoliosis, and other adverse consequences. Nonoperative treatment with physiotherapy can be attempted before surgery is considered but it usually fails. Operative treatment is the gold standard method of treatment for all established cases of GMC, and the clinical outcome is exceptionally good. However, different surgical methods should be selected carefully, and imaging examinations are crucial to making the correct treatment plan.^[7]^ Imaging examination shows the extent and severity of the disease and helps guide surgical treatment.^[11–16]^

However, few reports on the imaging presentation of GMC were available. X-ray examination mainly revealed an increased neck-shaft angle and center-edge angle, and decreased acetabular-head index and acetabular index.^[11,12]^ Radiographic signs only suggested the diagnosis of this disease but could not show the extent of affected muscle lesions. Computed tomography (CT) and magnetic resonance imaging (MRI) scans could directly determine the anatomical structure, location, extent, and severity of the disease, thus providing a diagnosis of the disease and a reliable surgical plan. Wang et al studied CT images of 61 patients with GMC and concluded the following as the main manifestations:^[13]^ reduced gluteal muscle volume, calcification and necrosis in injection sites, striped crispation of fascia, and widened gluteal muscle clearance. However, some scholars believed that the soft-tissue resolution of CT was poor, and the contracture strips were not display well. Relatively, MRI had a high resolution of soft tissue and could be multiazimuth, multisequence, and multiangle imaging. The MRI quality of contracture strips was clearer than that of the CT. Chen et al described MRIs in GMC and divided them into primary and secondary features.^[14]^ The primary manifestations were: atrophy of the gluteal muscle and intramuscular fibrotic cord. The secondary imaging findings were as follows: medial posterior displacement of the iliotibial tract, medial retraction of the affected gluteus maximus muscle, depressed groove at the muscle–tendon junction, and external rotation of the proximal femur. The characteristic fibrotic cord manifested low-signal intensity on all sequences and was most obvious on fat-suppressed images. The coronal section could show its movement.

For the past few decades, the conventional GSUS and Doppler US techniques have been routinely used in daily radiology practices for evaluating various traumatic and pathologic conditions of several musculoskeletal tissues, with results comparable to those of MRI.^[17]^ A small number of reports were available on US diagnosis of the disease.^[15,16]^ Compared with CT and MRI, US was simple, rapid, and economical with no radiation exposure. The US characteristics of non-GMC muscles were as follows. In the longitudinal view, the parallel hypoechoic arrays of the gluteus maximus were surrounded by hyperechoic areas of muscle and fascia membrane tissue, similar to a feather or spindle. In the transverse view, the muscle fibers appeared round, convex, or irregular (Fig. 4A and B). However, the 2-dimensional sonogram of GMC showed thinning of the affected muscle and presence of hyperechoic bands within the muscle. Statistical analysis showed that the presence of the contracture strips was more significant for the diagnosis of the disease. The sensitivity and specificity were 88.9% and 83.3%, respectively.^[16]^ In the 3 cases, we found the gluteus maximus muscle thinning and intramuscular cord-like zone of strong echo, which was similar to the those reported in previous studies.

Above all, the contracture strips are a characteristic change in GMC. The CT, MRI, and US have reported the signs,^[13–16]^ but no study has reported on the hardness of the contracture strips. Promising results have been obtained using SWE for evaluating several traumatic and pathologic conditions of various musculoskeletal soft tissues.^[18]^ In this study, the stiffness of the contracture strips of 3 patients was measured quantitatively by SWE, and was compared with that of the gluteal muscle of healthy individuals in the same position, and a significant difference was found. In patients with GMC, the shear-wave elastogram of the contracture strips was uneven blue-green, orange-yellow, or red, while it was mostly uniform light blue in the same position in healthy individuals (Fig. 4C and D). Whether in the transverse or longitudinal section, the shear-wave velocity in the contracture strips was greater than that in the normal gluteal muscle. The average of the mean shear-wave velocity of the contracture strips in the 6 sides of the hip of the 3 patients was 7.23 m/s (vertical profile). The average of the mean shear-wave velocity of the gluteal muscle in 1 healthy control in the same position was 1.84 m/s (vertical profile). This was found to be related to the histologic components of the contracture strips. Microscopic examination confirmed that the contracture strips consisted of dense collagen fibers and slender spindle-shaped fibroblasts were scattered in the thick collagen bundles and ran parallel to the collagen fibers, similar to the structure of tendons. This might explain the higher stiffness measurements of the contracture strips than those of the normal gluteus muscle.

In this study, the stiffness of the contracture strips was different between transverse and longitudinal sections. The stiffness measurements of the contracture strips in the longitudinal section were found to be higher than those in the corresponding transverse section. The reason may be the longitudinal arrangement of the fibrous tissue of the contraction strips. When the ultrasonic beam was parallel to the fibrous tissue, the measurement results were more accurate. Different measurements of stiffness at different sections indicated that the contracture strips were composed of anisotropic tissue.

We found that the stiffness measurements of the contracture strips increased as the signs and symptoms aggravated. In the 3 patients, as the limitation of the hip joint function aggravated, the angle of passive adduction in hip flexion decreased, and the average of the mean shear-wave velocity of the contracture strips increased (vertical profile). Therefore, we speculate the hardness of the contracture band was related to the severity of local lesions. However, this phenomenon was not found in the transverse section.

The gross pathologic examination of the 3 cases showed the involvement of piriformis muscle, but we did not detect imaging changes by US. This may be due to the relative position of the muscles. The gluteus maximus is superficial, so that the abnormal echo can be easily observed. However, the piriformis muscle is more deeply located; therefore, it is not easily detected by the transducer.

## 5 Conclusion

We found that the stiffness of the contracture strips was higher than that of the normal gluteus muscle. The results of this study suggested that the stiffness of the contracture strips could predict the severity of GMC. However, more research is needed to prove this finding. Moreover, the use of longitudinal stiffness measurements was recommended to assess the severity of GMC because of the anisotropic characteristics of the contracture strips.

## Acknowledgment

The authors thank Editage (www.editage.com) for English language editing.

## Author contributions

**Conceptualization:** Li Qiu.

**Data curation:** Ruiqian Guo.

**Writing – original draft:** Ruiqian Guo.

**Writing – review & editing:** Xi Xiang, Li Qiu.
